# A fast forward 3D connection algorithm for mitochondria and synapse segmentations from serial EM images

**DOI:** 10.1186/s13040-018-0183-7

**Published:** 2018-11-05

**Authors:** Weifu Li, Jing Liu, Chi Xiao, Hao Deng, Qiwei Xie, Hua Han

**Affiliations:** 1 Faculty of Mathematics and Statistics, Hubei University, 368 Youyi Road, Wuhan, 430062 China; 2 Institute of Automation, Chinese Academy of Sciences, 95 Zhongguancun East Road, Beijing, 100190 China; 3 Faculty of Information Technology, Macau University of Science and Technology, Avenida Wai Long,Taipa, Macau, China; 4 Data Mining Lab, Beijing University of Technology, 100 Ping Le Yuan, Beijing, 100124 China; 5 Research Base of Beijing Modern Manufacturing Development, 100 Ping Le Yuan, Beijing, 100124 China; 6 Center for Excellence in Brain Science and Intelligence Technology Shanghai Institutes for Biological Sciences, Chinese Academy of Sciences, 320 Yue Yang Road, Shanghai, 200031 China; 7 School of Future Technology, University of Chinese Academy of Sciences, 19 Yuquan Road, Beijing, 100190 China

**Keywords:** 3D connection, EM images, *Bwconncomp*, Mitochondria, Synapse

## Abstract

**Background:**

It is becoming increasingly clear that the quantification of mitochondria and synapses is of great significance to understand the function of biological nervous systems. Electron microscopy (EM), with the necessary resolution in three directions, is the only available imaging method to look closely into these issues. Therefore, estimating the number of mitochondria and synapses from the serial EM images is coming into prominence. Since previous studies have achieved preferable 2D segmentation performance, it holds great promise to obtain the 3D connection relationship from the 2D segmentation results.

**Results:**

In this paper, we improve upon Matlab’s function *bwconncomp* and propose a fast forward 3D connection algorithm for mitochondria and synapse segmentations from serial EM images. To benchmark the performance of the proposed method, two EM datasets with the annotated ground truth are produced for mitochondria and synapses, respectively. Experimental results show that the proposed method can achieve the preferable connection performance that closely matches the ground truth. Moreover, it greatly reduces the computational burden and alleviates the memory requirements compared with the function *bwconncomp*.

**Conclusions:**

The proposed method can be deemed as an effective strategy to obtain the 3D connection relationship from serial mitochondria and synapse segmentations. It is helpful to accurately and quickly quantify the statistics of the numbers, volumes, surface areas, and lengths, which will greatly facilitate the data analysis of neurobiology research.

## Background

Recently, due to the rapid development of neuroscience, considerable attention has been paid for the statistics of the numbers, volumes, surface areas, and lengths of sub-cellular structures in the brain, allowing neuroscientists to compare these objects in healthy animals and those with degenerative brain diseases [1]. It acts as an important role in studying the sub-cellular structures and their implied behaviors of nervous systems [2]. Among these structures, mitochondria and synapses are of particular interest to neuroscience [3]. Indeed, mitochondria, known as the powerhouse of the cell, have been proven to carry out all types of important cellular functions by producing the overwhelming majority of cellular adenosine triphosphate (ATP) [4]. Meanwhile, they also take substantial responsibility for the regulation of cellular life and death, as well as disease states. For example, mitochondrial dysfunction has been directly linked to the aging process, which is the largest single risk factor for Alzheimer Disease [5, 6]. In addition, synapses, known as the information transmitters, permit a neuron to pass an electrical or chemical signal to another neuron in the mammalian nervous system. Mounting evidence also indicates that synaptic plasticity has a close link with learning and memory. To be specific, sensory experience, motor learning and aging are found to induce alterations in presynaptic axon boutons and postsynaptic dendritic spines [7, 8]. Consequently, the quantification of mitochondria and synapses is of great significance to the prevention and treatment of brain diseases.

The above researches provide the motivation for looking closely into these issues in a nervous system, and image analysis techniques as an important approach are widely adopted. For image acquisition, electron microscopy (EM), with sufficiently high resolution on the nanoscale, can provide not only the details of intra-cellular structures but also the synapses and gap junctions. In particular, focused ion beam scanning electron microscopy (FIB-SEM) [9] can provide an isotropic resolution up to 5 *nm* in three directions, and automated tape-collecting ultramicrotome scanning electron microscopy (ATUM-SEM) [10] can offer an anisotropic voxel (4 *nm*× 4 *nm*× 30 *nm*) with a low resolution in the *z* direction. However, the FIB-SEM technique is limited to a small volume while the ATUM-SEM technique does not suffer from the limitation and can be applied to a large volume for the statistics and analysis [11]. To extract the invaluable structural information (mitochondria and synapses) from the serial EM images, substantial effort has been recently put into developing specialized algorithms for accurate segmentation of mitochondria and synapses. For some representative results, Lucchi et al. [12] clustered groups of similar voxels into regularly spaced supervoxels and incorporated mitochondrial shape features to an automated graph partitioning scheme for segmentation. On this base, they [13] introduced context-based features and modelled mitochondrial membranes for improvement. Staffler et al. [14] presented a method for automated detection of synapses, which focused on classifying borders between neuronal processes as synaptic or non-synaptic. Neila et al. [3] proposed an automated approach for both mitochondria and synapses that involved anisotropy-aware regularization via conditional random field inference and surface smoothing techniques to improve the segmentation and visualization. Moreover, due to the powerful representation capability of deep neural network, Xiao et al. [15] put forward a fusion fully convolutional network for mitochondrial segmentation and a fully connected conditional random field to optimize the segmentation results. Santurkar et al. [16] took a compositional approach to segment synapses by training lighter networks to model the simpler marginal distributions of membranes, clefts and vesicles.

Although above approaches have demonstrated preferable segmentation performance, it seems that the connection mode from serial binary segmentation results has not received enough attention in the anisotropic case. A recent research, Neila et al. [3] adopted an approximate, simple solution under the assumption that each connected component of the segmentation is one structure, and they also pointed out that estimating the number of structures from the segmentation results is still an open problem with plenty of ongoing research. Consequently, our aim in this paper is to develop a fast and effective 3D connection algorithm based on these segmentation results. The main contributions can be roughly grouped in two different directions.

- **Methods:** Since the shapes of mitochondria and synapses have great differences in EM images, we propose a new similarity indicator that takes the shape into consideration. It could accurately measure the probability that the segmentation results belong to the same 3D mitochondrion or 3D synapse. In addition, we propose a forward connection mode that can effectively handle the problems of split and merge. This connection mode can be generalized to serial detection results, but not limited to serial segmentation results.
- **Data:** We benchmark the performance of the proposed method against a previously unreleased corpus of manually annotated data. The corpus consists of two EM datasets acquired by the ATUM-SEM technique and the ground truth of mitochondria and synapses are manually annotated by 2-3 independent labelers, respectively. Both the EM datasets and manual annotations are released to the community providing a valuable tool for benchmarking.

The subsequent sections of this study present the detailed information about the datasets, the proposed connection algorithm, the experimental results, the meaningful discussions and conclusions.

## Materials

In this paper, the biological specimens were selected from mouse cortex and the ATUM-SEM technique was adopted for image acquisition. The datasets were collected from a water bath using a custom designed tape-collection conveyor belt in the Institute of Neuroscience, Chinese Academy of Sciences, where several slices with thicknesses of more or less 50 *nm* were cut automatically. Next, these sections were imaged through SEM (Zeiss Supra55) in the Institute of Automation, Chinese Academy of Sciences, where the pixel size was set at 2 *nm* and the dwell time was set at 2 *μ*s. Since the datasets acquired by the ATUM-SEM technique were unregistered, the image registration method applied in [17] was adopted in this paper. After registration, two ATUM-SEM datasets are used to construct the corresponding databases for mitochondria and synapses, respectively.

### Mitochondria dataset

**Fig. 1:**
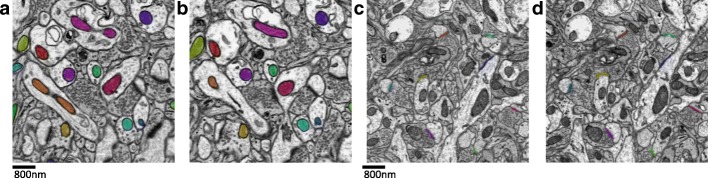
**a** and **b**: adjacent ATUM-SEM images with the mitochondria annotated by different colors; **c** and **d**: adjacent ATUM-SEM images with the annotated synapses

The mitochondria dataset consists of 31 slices with a resolution of 2×2×50*nm*^3^/*voxel*, and each slice has a size of 8416×8624 [18]. The ground truth were prepared via the hand segmentation outlining the mitochondrial membrane by two labelers with cross validation. A total of 473 mitochondria including the incomplete ones were annotated with the plugin TrakEm2 in software ImageJ [19]. Figure 1a-b present the acquired images and annotated mitochondria in the adjacent slices, where different mitochondria are represented by different colors. To accelerate the neurobiology research, we share the mitochondria dataset and the annotated ground truth publicly available in the website^1^.

### Synapse dataset

Since the synapses are more sparsely distributed than the mitochondria in the biological tissue, we need a larger volume for statistics and analysis. Here, the synapse dataset consists of 178 slices with the resolution 2×2×50*nm*^3^/*voxel* and size 8576×7616 [15]. The ground truth were prepared via the hand segmentation outlining the synaptic junctions by three labelers with cross validation. A total of 1230 synapses were annotated by different colors. Figure 1c-d present the acquired images and annotated synapses in the adjacent slices. Also, the synapse dataset and the annotated ground truth are provided publicly available in the website^2^.

It is worthwhile to emphasize that creating such two databases requires a considerable amount of human effort, and it is a considerably time-consuming process which also justifies that previous endeavors on computerized segmentation are of great significance for the neurobiology research.

## Methods

As mentioned above, the EM images with high resolution will inevitably produce large data even at small neural circuit. From a practical point of view, it is time consuming and memory consuming to directly measure the similarity of segmentations in the adjacent slices. Therefore, we propose a fast coarse-to-fine connection algorithm instead. The main motivation is given by the following axiom.

*Axiom 1:* Let *s*_1_ and *s*_2_ be the segmentations, and *d*_1_ and *d*_2_ be the regions satisfying *s*_1_⊂*d*_1_ and *s*_2_⊂*d*_2_. Then if $d_{1}\bigcap d_{2}=\emptyset $, $s_{1}\bigcap s_{2}=\emptyset $.

This axiom indicates that we can use the bounding boxes containing the segmentations for screening to reduce the computation cost. The proposed connection algorithm is divided into four steps: coarse screening, validation, fine connection and skip connection. The detailed procedures are summarized in the following subsections.

### Coarse screening

On basis of the 2D segmentation results, we first obtain the corresponding bounding boxes by the Matlab’s function *regionprops*. Assume that there are *n* slices and each slice has *k*_*i*_ segmentations *i*=1,2,⋯,*n*, and the *p*th segmentation in the *i*th slice is denoted by matrix $s^{i}_{p}$ and its bounding box is denoted by coordinate vector $X^{i}_{p}$, the coarse similarity $c_{pq}^{i}$ of segmentations $s^{i}_{p}$ and $s^{i+1}_{q}$ is measured by the Intersection-over-Union (IoU) of bounding boxes $X^{i}_{p}$ and $X^{i + 1}_{q}$:

1 $$ c_{pq}^{i}:=\text{IoU}\left(X^{i}_{p},X^{i+1}_{q}\right)=\frac{A\left({X^{i}_{p}}\cap X^{i+1}_{q}\right)}{A\left({X^{i}_{p}}\cup X^{i + 1}_{q}\right)}.  $$

Here, $A\left ({X^{i}_{p}}\cap X^{i+1}_{q}\right)$ and $A\left ({X^{i}_{p}}\cup X^{i+1}_{q}\right)$ denote the areas of the intersection and union of $X^{i}_{p}$ and $X^{i + 1}_{q}$, respectively. Since the intersection is empty with high probability when $X^{i}_{p}$ and $X^{i+1}_{q}$ belong to the different 3D structures, it is clear that $c_{pq}^{i},~p=1,\cdots,k_{i},~q=1,\cdots,k_{i+1}$ are almost zeros. Therefore, we can use a sparse matrix *C*^*i*^ to denote the coarse connection relation between the *i*th slice and the *i*+1th slice as follows:

2 $$ C^{i} = \left({\begin{array}{cccc} {c_{11}^{i}}&{c_{12}^{i}}& \cdots &{c_{1k_{i+1}}^{i}}\\ {c_{21}^{i}}&{c_{22}^{i}}& \cdots &{c_{2k_{i+1}}^{i}}\\ \vdots & \vdots & \ddots & \vdots \\ {c_{k_{i}1}^{i}}&{c_{k_{i}2}^{i}}& \cdots &{c_{k_{i}k_{i+1}}^{i}} \end{array}}\right),~ i=1,2,\cdots,n-1.  $$

According to axiom 1, the position information provided by the bounding box is only a necessary condition, it may produce superfluous connections. As illustrated in Fig. 2c, the connection matrix cannot accurately reflect the connection relationship between the segmentations in Fig. 2a-b. Note that a larger similarity $c_{pq}^{i}$ means a higher probability that the segmentations $s^{i}_{p}$ and $s^{i+1}_{q}$ belong to the same 3D structure. Two thresholds 0≤*T*_*l*_≤*T*_*h*_≤1 are adopted to judge whether the $s^{i}_{p}$ and $s^{i+1}_{q}$ are connected. Three cases are listed as below:

1. If $c_{pq}^{i}\in [T_{h},1]$, there exists a connection between the $s^{i}_{p}$ and $s^{i+1}_{q}$;

**Fig. 2:**
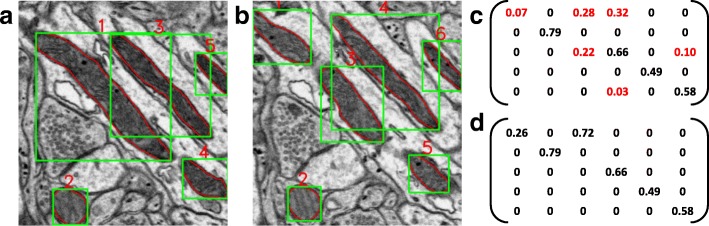
A schematic diagram of coarse-to-fine similarity measure. **a** and **b** present the original images in the adjacent slices, where the segmentation results are outlined by red curves and the corresponding bounding boxes are depicted by green boxes. **c** and **d** present the coarse connection matrix and the updated matrix
2. If $c_{pq}^{i}\in [0, T_{l})$, there exists no connection between the $s^{i}_{p}$ and $s^{i+1}_{q}$. Set $c_{pq}^{i}=0$;
3. If $c_{pq}^{i}\in [T_{l}, T_{h})$, there may be a connection between the $s^{i}_{p}$ and $s^{i+1}_{q}$, which needs to be validated as marked in red in Fig. 2c (*T*_*l*_=0.01 and *T*_*h*_=0.4 are used).

### Validation

In this subsection, we utilize the segmentation information to validate the similarities in case (3). According to the coordinate vectors $X^{i}_{p}$ and $X^{i + 1}_{q}$, we can find a minimum image domain *I* that contains the corresponding segmentations, namely, two binary images denoted by $I^{i}_{p}$ and $I^{i + 1}_{q}$, respectively. On this base, we update the similarity $c_{pq}^{i}$ of the segmentations $s^{i}_{p}$ and $s^{i+1}_{q}$ by considering the invariable position term $P\left (s^{i}_{p},s^{i + 1}_{q}\right)$ and the variational shape term $S\left (s^{i}_{p},s^{i + 1}_{q}\right)$:

3 $$ c_{pq}^{i} = \frac{P^{2}\left(s^{i}_{p},s^{i + 1}_{q}\right)+\lambda\cdot S^{2}\left(s^{i}_{p},s^{i + 1}_{q}\right)} {1+\lambda}.  $$

Here, *λ*≥0 is a regularization parameter for balance. $P\left (s^{i}_{p},s^{i + 1}_{q}\right)$ characterizes the invariance of segmentations $s^{i}_{p}$ and $s^{i+1}_{q}$, and is defined by the IoU of the corresponding binary images $I^{i}_{p}$ and $I^{i + 1}_{q}$:

4 $$ P\left(s^{i}_{p},s^{i + 1}_{q}\right)=\text{IoU}\left(I^{i}_{p},I^{i+1}_{q}\right).  $$

In contrast, $S\left (s^{i}_{p},s^{i + 1}_{q}\right)$ characterizes the variability of segmentations $s^{i}_{p}$ and $s^{i+1}_{q}$. Assume that the essential transformation $h: s^{i}_{p}\longrightarrow s^{i + 1}_{q}$ has the form *h*=*h*_*α*_*h*_*β*_, *h*_*α*_(*x*)=*αx* (scaling), and *h*_*β*_(*x*^′^)=*x*^′^+*β* (translation) [20]. Then given a set of *H* transformations $\mathcal {H}=\{h_{1}, h_{2}, \cdot \cdot \cdot, h_{H}\}$, $S\left (s^{i}_{p},s^{i + 1}_{q}\right)$ is defined by:

5 $$\begin{array}{*{20}l} S\left(s^{i}_{p},s^{i + 1}_{q}\right)=\max\left\{\left.P\left(h_{k}\left(s^{i}_{p}\right),s^{i + 1}_{q}\right)\right|k=1,2,\cdots,H\right\}. \end{array} $$

After updating all the similarities in case (3), we can obtain new connection matrices *C*^*i*^,*i*=1,2,⋯,*n*−1. For example, the similarities marked in red in Fig. 2c are validated and the updated matrix is shown in Fig. 2d. It is clear that the updated matrix effectively eliminates the false connections. Then, another threshold *T*_*s*_∈[0,*T*_*h*_) is adopted to determine the fine connection matrices:

6 $$ B^{i}=C^{i}>T_{s}, i=1,2,\cdots,n-1.  $$

### Fine connection

For each binary connection matrices *B*^*i*^,*i*=1,2,⋯,*n*−1, the sum of the *p*th row $R_{p}^{i}~(p=1,2,\cdots, k_{i})$ implies that $s_{p}^{i}$ connects with $R_{p}^{i}$ segmentations in the *i*+1th slice, and the sum of the *q*th column $N_{q}^{i+1}~\left (q=1,2,\cdots, k_{i+1}\right)$ implies that $N_{q}^{i+1}$ segmentations in the *i*th slice connects with $s_{q}^{i+1}$. Based on this fact, we propose a forward connection mode instead of the iterative bidirectional connection mode in [21]. The details are divided into three steps.

**Fig. 3:**
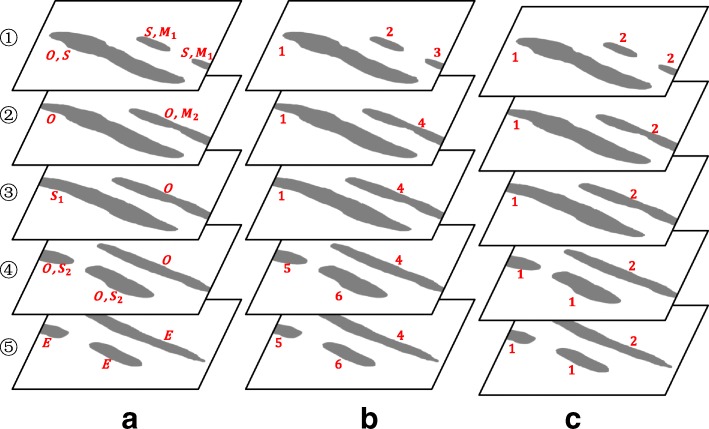
A schematic diagram of the proposed connection mode. **a** assigning several categories to each segmentation; **b** assigning an initial label to each segmentation; **c** reassigning the same label to the segmentations in case of split and merge

Firstly, we assign several categories to each segmentation according to the $R_{p}^{i}$ and $N_{q}^{i+1}$ as illustrated in Fig. 3a. These categories include *One-to-one* (*O*), *Start* (*S*), *End* (*E*), *Split*_1_ (*S*_1_), *Merge*_1_ (*M*_1_), *Split*_2_ (*S*_2_) and *Merge*_2_ (*M*_2_). For clarity of presentation, we provide a simplified matrix of *B*^*i*^ in Table 1. The five general connection cases are listed as follows: (1) since $R_{1}^{i}=N_{1}^{i+1}=1$, we assign *O* to the first segmentation in the *i*th slice; (2) since $R_{2}^{i}=0$, we assign *E* to the second segmentation in the *i*th slice; (3) since $N_{4}^{i+1}=0$, we assign *S* to the fourth segmentation in the *i*+1th slice; (4) since $R_{3}^{i}=2$ and $N_{2}^{i+1}=N_{3}^{i+1}=1$, we assign *S*_1_ to the third segmentation in the *i*th slice and *S*_2_ to the second and the third segmentations in the *i*+1th slice; (5) since $R_{4}^{i}=R_{5}^{i}=1$ and $N_{5}^{i+1}=2$, we assign *M*_1_ to the fourth and the fifth segmentations in the *i*th slice and *M*_2_ to the fifth segmentation in the *i*+1th slice. Moreover, *S* is assigned to each segmentation in the first slice and *E* is assigned to each segmentation in the final slice. It should be noted that each segmentation may have two or more categories as shown in Fig. 3a.

**Table 1:** A simplified matrix of *B*^*i*^ with five general connection cases

Category	−−	*Split*_2_	*Split*_2_	*Start*	*Merge*_2_
*One-to-one*	1	0	0	0	0
*End*	0	0	0	0	0
*Split*_1_	0	1	1	0	0
*Merge*_1_	0	0	0	0	1
*Merge*_1_	0	0	0	0	1

Secondly, we assign an initial label to each segmentation according to the categories as illustrated in Fig. 3b. Specifically, we first denote the segmentation sets with category *O*, with category *S*, with category *E*, with category *S*_1_, with category *M*_1_, with category *S*_2_ and with category *M*_2_ in the *i*th slice as *O*^*i*^, *S*^*i*^, *E*^*i*^, $S_{1}^{i}$, $M_{1}^{i}$, $S_{2}^{i}$ and $M_{2}^{i}$, respectively. Then we begin with each segmentation $s\in S^{i}\bigcup S_{2}^{i}\bigcup M_{2}^{i}, i=1,2,\cdots n,$ and assign a unique label *j* to it. If *s*∈*O*^*i*^, find the connected segmentation *s*_1_ in the *i*+1 slice by *B*^*i*^ and assign the same label *j* to *s*_1_. Set *s*=*s*_1_,*i*=*i*+1, and repeat above steps until *s*∉*O*^*i*^.

Thirdly, we reassign the same label to the segmentations in case of split and merge as illustrated in Fig. 3c. Specifically, for each segmentation $s_{1} \in M_{1}^{i} \bigcup S_{1}^{i}, i=1,2,\cdots n-1$, we first obtain the label *j*_1_ of *s*_1_ and the label *j*_2_ of the connected segmentation $s_{2}\in M_{2}^{i+1} \bigcup S_{2}^{i+1}$ in the *i*+1 slice by *B*^*i*^. Then, we reassign the label min{*j*_1_,*j*_2_} to these segmentations with labels *j*_1_ and *j*_2_.

#### **Remark 1**

By this connection mode, we assign different labels to these segmentations. When *λ*=0, $\phantom {\dot {i}\!}T_{s}=T_{d_{2}}=0$, the coarse-to-fine connection method has the same performance as the Matlab’s function *bwconncomp*, which judges whether the segmentation results are connected by the specified connectivity for the connected components.

### Skip connection

The matrices *B*^*i*^,*i*=1,2,⋯,*n*−1, only characterize the connection relationship in the adjacent slices. However, it is usually hard to prevent wrinkle and damage from sample preparation and imaging in practice. Additionally, a minority of objects are difficult to be identified because they sometimes do not exhibit their typical characteristics on a certain slice. Therefore, the connection relationship in the skipped slices should also be considered. Based on the above considerations, we first calculate the coarse connection matrices as:

7 $$ C^{i2} = \left({\begin{array}{cccc} {c_{11}^{i2}}&{c_{12}^{i2}}& \cdots &{c_{1k_{i+2}}^{i2}}\\ {c_{21}^{i2}}&{c_{22}^{i2}}& \cdots &{c_{2k_{i+2}}^{i2}}\\ \vdots & \vdots & \ddots & \vdots \\ {c_{k_{i}1}^{i2}}&{c_{k_{i}2}^{i2}}& \cdots &{c_{k_{i}k_{i+2}}^{i2}} \end{array}} \right), i=1,2,\cdots,n-2,  $$

where the $c_{pq}^{i2}, p=1,2,\cdots,k_{i},~q=1,2,\cdots,k_{i+2}$ is the coarse similarity of $s^{i}_{p}$ and $s^{i+2}_{q}$. Then, we focus on each pair of segmentations $s^{i}_{p}\in E^{i}$ and $s^{i+2}_{q}\in S^{i+2}$. If $c_{pq}^{i2}>0$, update the $c_{pq}^{i2}$ by formula (3) and judge the connectivity by threshold *T*_*s*_. If $c_{pq}^{i2}>T_{s}$, reassign a new label min{*j*_1_,*j*_2_} to the segmentations with labels *j*_1_ and *j*_2_, where *j*_1_ and *j*_2_ are the labels of $s^{i}_{p}$ and $s^{i+2}_{q}$, respectively.

The proposed method is sketched in the following Algorithm 1. By using the proposed algorithm, we divide the whole segmentations into several disjoint sets, which satisfies that the segmentations in the same set belong to the same 3D object while these in the different sets belong to different 3D objects.


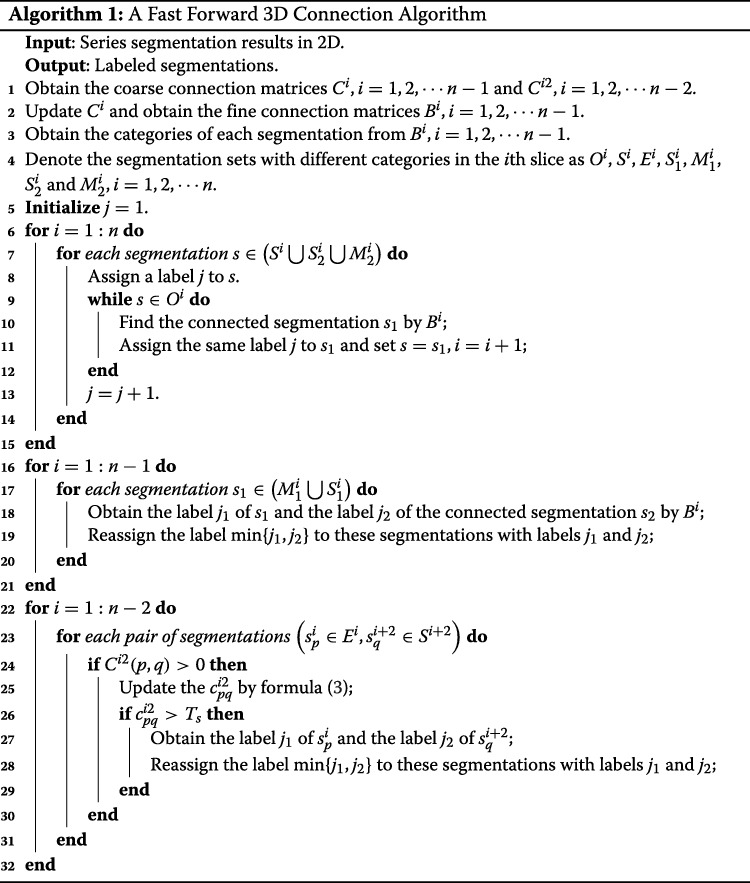


## Results

In this section, we conduct several experiments to evaluate the performance of the proposed algorithm on the above mentioned datasets. The connection capacity is measured by two fundamental performance indicators, *split error* and *merge error* [22]. Here, the *split error* means that a true 3D object is regarded as several 3D objects, which often occurs when large objects sometimes split into two or more connected components. In contrast, the *merge error* means that several true 3D objects are regarded as a whole 3D object and it will occur when a group of structures close to each other often merge in a single connected component [3].

### Performance comparison

**Fig. 4:**
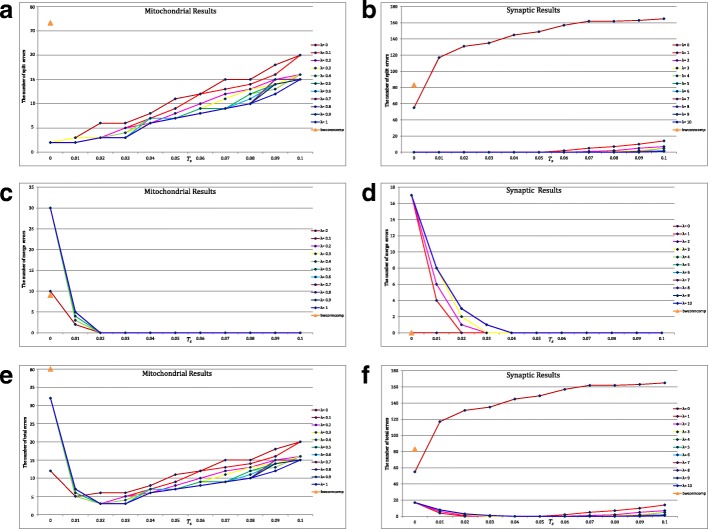
**a** and **b** the number of split errors of the function *bwconncomp* and the proposed method at varying thresholds *λ* and *T*_*s*_; **c** and **d** the number of merge errors; **e** and **f** the number of total errors

The parameters in the proposed algorithm have a huge impact on the final connection performance. For example, a larger *T*_*l*_ tends to produce split errors and a smaller *T*_*h*_ tends to produce merge errors. To guarantee a better connection performance, a relatively small threshold *T*_*l*_=0.01 and a relatively large threshold *T*_*h*_=0.4 are chosen for coarse screening although it will take more time for verification. The choice of parameter *λ* depends on the size of the segmentation results. In the mitochondria experiments, since the diameter of mitochondria is commonly between 0.75 and 3 *μm* [23], the segmentation results that belong to the same 3D mitochondrion usually have large overlap in the adjacent slices. Then *λ* is set from 0 to 1 with step size 0.1 to satisfy that the position term in (3) is the dominant contribution for the similarity measure. In the synapse experiments, the mean cleft width of wild-type synapses is 22±0.5 *nm* between the pre- and postsynaptic neurons [24, 25]. Due to the offsets and differences in the adjacent slices, the segmentation results that belong to the same 3D synapse usually have small or even no overlaps. Then *λ* is set from 0 to 10 with step size 1 to satisfy that the shape term in (3) is the dominant contribution. Meanwhile, we adjust the threshold *T*_*s*_ from 0 to 0.1 with step size 0.01. The number of split errors, the number of merge errors and the number of total errors of the proposed method at varying thresholds *λ* and *T*_*s*_ are illustrated in Fig. 4a-b, c-d and e-f, respectively, where the results of the function *bwconncomp* are also presented as baseline for comparison. Several useful conclusions can be drawn from Fig. 4.

**Firstly, the step ‘skip connection’ in the proposed method is capable of reducing the number of split errors.** As mentioned in Remark 1, the function *bwconncomp* is a special case of the proposed method. Specifically, adding the step ‘skip connection’ to the function *bwconncomp* has the same performance as the proposed method at *λ*=0 and *T*_*s*_=0. As shown in Fig. 4a-b, the step ‘skip connection’ can greatly reduce the split errors compared with the function *bwconncomp*. That is because the function *bwconncomp* only finds the connected components in the adjacent slices and splits a 3D object into two connected components when the information is missing. In contrast, our approach avoids this shortcoming by the step ‘skip connection’.

**Secondly, the suitable parameters*λ* and*T***_***s***_** play an important role in the final connection performance.** The regularization parameter *λ* is used for controlling the significance of the shape term in (3). Usually, a large *λ* is prone to produce merge errors while a small *λ* usually leads to split errors. The suitable choice of *λ* plays an important role in the balance. As shown in Fig. 4a-b, the choice *λ*>0 significantly reduces the number of split errors compared with *λ*=0 (function *bwconncomp*), especially in the synapse dataset. It demonstrates the superiority of the proposed similarity indicator. In addition, as another threshold *T*_*s*_ for determining the fine connection, a large *T*_*s*_ tends to produce split errors while a small *T*_*s*_ tends to produce merge errors. Similarly, compared with *T*_*s*_=0 (function *bwconncomp*) in Fig. 4c-d, the choice *T*_*s*_>0 greatly reduces the number of merge errors. Taking these factors into consideration, the suggested parameters are *λ*∈(0.4,0.6),*T*_*s*_∈(0.02,0.03) for the mitochondria dataset and *λ*∈(1,3),*T*_*s*_∈(0.03,0.05) for the synapse dataset, respectively as shown in Fig. 4e-f.

**Thirdly, the proposed method achieves the near-human performance in obtaining the 3D connection relationship.** Given the suggested parameters, there are only three split errors on the mitochondria dataset, and the optimal case without split error and merge error is achieved on the synapse dataset. Note that the proposed method achieves the optimal performance for a wide range of thresholds in Fig. 4e-f. The robustness is demonstrated.

**Fig. 5:**
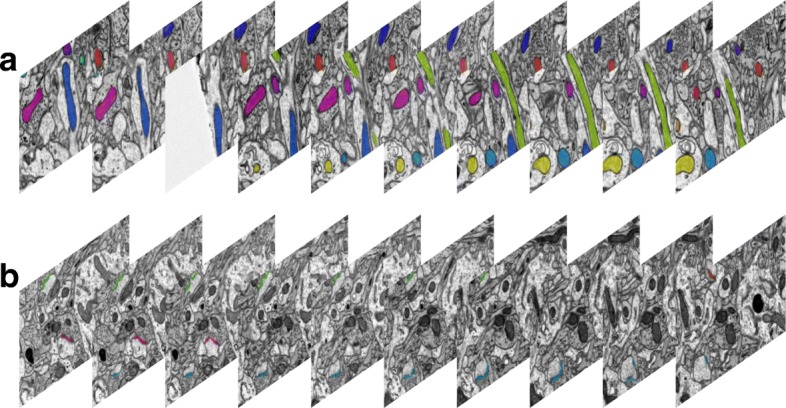
**a** and **b** the specific connection results of the proposed method

To have a visual presentation, we provide the specific connection results of the proposed method on the mitochondria dataset and the synapse dataset in Fig. 5, where these segmentations that belong to the same 3D object are described by the same colors. It is clear that the proposed connection algorithm can effectively handle the problems of split and merge (Fig. 5).

### Running time comparison

**Fig. 6:**
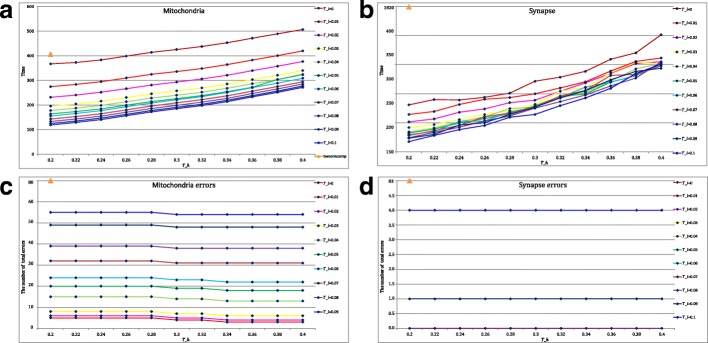
**a** and **b** the time consumption of the function *bwconncomp* and the proposed method at varying thresholds; **c** and **d** the corresponding connection performance

As mentioned in the previous subsection, the choices of *T*_*l*_ and *T*_*h*_ are not only related to the connection performance but also the time consumption. In this subsection, we first preset the optimal parameters *T*_*s*_=0.03, *λ*=0.5 for the mitochondria experiments and *T*_*s*_=0.03, *λ*=2 for the synapse experiments. Then, the threshold *T*_*l*_ from 0 to 0.1 with step size 0.01 and threshold *T*_*h*_ from 0.2 to 0.4 with step size 0.02 are adjusted to estimate the time consumption. Figure 6a-b present the time consumption of the function *bwconncomp* and the proposed method at varying thresholds for comparison. Meanwhile, the corresponding connection performance is also provided for referencing in Fig. 6c-d. Several useful conclusions can be drawn from Fig. 6.

**Firstly, the suitable parameters*T***_***h***_** and*T***_***l***_** reduce the time consumption as well as keep the preferable performance.** From Fig. 6, it is obvious that a larger *T*_*h*_ and a smaller *T*_*l*_ will take more time for computation on the both datasets. Note that the number of errors does not decrease when *T*_*h*_≥0.34,*T*_*l*_≤0.01 for the mitochondria dataset, and *T*_*h*_≥0.2,*T*_*l*_≤0.07 for the synapse dataset, the suggested parameter interval are *T*_*h*_∈(0.34,0.4),*T*_*l*_∈(0,0.01) and *T*_*h*_∈(0.2,0.3),*T*_*l*_∈(0,0.07), respectively. For a detailed representation, the time consumption of the function *bwconncomp* and the proposed method at several suggested parameters are provided in Table 2.

**Table 2:** The time consumption of the function *bwconncomp* and the proposed method at several suggested parameters

Mitochondria dataset		Synapse dataset	
*bwconncomp*	398s	*bwconncomp*	1815s
*T*_*h*_=0.40,*T*_*l*_=0.01	419s	*T*_*h*_=0.26,*T*_*l*_=0.01	240s
*T*_*h*_=0.38,*T*_*l*_=0.01	400s	*T*_*h*_=0.24,*T*_*l*_=0.01	231s
*T*_*h*_=0.36,*T*_*l*_=0.01	382s	*T*_*h*_=0.22,*T*_*l*_=0.01	219s
*T*_*h*_=0.34,*T*_*l*_=0.01	364s	*T*_*h*_=0.20,*T*_*l*_=0.01	214s

**Secondly, the proposed method is more computationally efficient than the function*bwconncomp*.** From Table 2, we can see that the proposed method only needs an average of 300s to obtain the connection results while the function *bwconncomp* needs 398s on the mitochondria dataset and 1815s on the synapse dataset. It is because the size of synapse dataset is 6 times larger than that of mitochondria dataset and the function *bwconncomp* obtains the connected components by handling with each pixel. However, the proposed method uses the thresholds *T*_*l*_ and *T*_*h*_ for coarse screening and only computes the region of interest instead of the whole image, which reduces the computation cost.

### Memory comparison

In this subsection, we present the memory requirement of the function *bwconncomp* and the proposed method in Table 3. It can be seen that the memory requirement of the proposed method is less than 1/6 of the function *bwconncomp* on the mitochondria dataset and approximately 1/10 of the function *bwconncomp* on the synapse dataset, respectively. It is mainly because the input of function *bwconncomp* must be the total serial segmentation results, i.e., the memory requirement is closely related to the data size. In contrast, the proposed method only needs to read two images repeatedly for calculating the connection matrices. The memory requirement is determined by these sparse connection matrices, which is independent of the data size. It indicates that the proposed method does not suffer from the common problem “Out of Memory” caused by large dataset.

**Table 3:** The memory requirement of the function *bwconncomp* and the proposed method

	Mitochondria dataset	Synapse dataset
*bwconncomp*	20.85 GB	44.02 GB
Our method	3.35 GB	4.41 GB

### Information sharing

All codes are written in Matlab Version R2016b (Math Works, Inc.). The mitochondria experiments are performed on a personal computer with an i7-4790 MQ 3.60 GHz Intel processor, 32 GB RAM and Windows operating system. Since the error “Out of Memory” happens when the function *bwconncomp* is tested on the synapse dataset, the synapse experiments are performed on a public server with an i7-4820 MQ 2.00 GHz Intel processor, 512 GB RAM and Windows operating system. The codes are available online^3^.

## Discussion

This present research is primarily motivated by the need to accurately obtain the statistics of mitochondria and synapses from serial EM images, which helps the neuroscientists to quickly quantify these objects in healthy and diseased animals. Since previous researches have achieved preferable performance on 2D segmentations [3, 15, 16, 18, 26], this paper has proposed a fast and effective method to obtain the 3D connection relation. To validate the effectiveness of the proposed method, we produce two ATUM-SEM datasets with the annotated ground truth for mitochondria and synapses, respectively. Experimental results show the superiority of the proposed method on the connection performance, running time and memory requirement over the function *bwconncomp*.

Because the proposed method only depends on the shapes and distribution of the objects, it indicates that the proposed method can also achieve a robust 3D connection performance for other subcellular structures, such as the endoplasmic reticulum, Golgi apparatus, and microtubules. Because all these structures are sparsely distributed in the EM images, relatively smaller parameters *T*_*l*_=0.01,*T*_*h*_=0.2 can be adopted for reducing the time consumption. Meanwhile, the shapes of endoplasmic reticulum and microtubules are narrow as the synapses, and the shapes of Golgi apparatus are elliptical as the mitochondria. The suggested parameters can be *λ*=2,*T*_*s*_=0.03 for the endoplasmic reticulum and microtubules, and *λ*=0.5,*T*_*s*_=0.02 for the Golgi apparatus.

Despite the promising results of the proposed method, several problems still deserve further research. The most important concern is that the connection performance usually relies heavily on the segmentation results. However, previous segmentation algorithms [3, 15, 16, 18, 26] almost focus on the lower pixel-wise prediction error (*pixel error*). Unfortunately, *pixel error* considers only whether or not a given pixel is correctly classified as the object, without concern for the ultimate effect on the connection performance. For example, expanding, shrinking or translating the object between two slices would not cause splits or mergers, but incur a large pixel error. Further, while a gap of even a single pixel in the object between two slices would cause a merge error, it might only incur a very small pixel error [27]. Thus, further research on segmentation algorithms should take other indicators such as the *rand error* [28], *warping error* [29] into consideration. In addition, a good connection method is expected to be more robust to different segmentation results. The generalization performance of the proposed method should be further validated on the results obtained by state-of-the-art segmentation algorithms. Future research will focus on using the 3D connection relation for optimizing the local misleading segmentation. As another concern, the effectiveness of the proposed method may owe to the characteristic that both the mitochondria and synapses are sparsely distributed in EM images. More future investigations along the present line will validate the generalization performance of the proposed method on the dense neuron segmentations. In addition, since it will yield more split errors and merge errors when the number of segments is large, some normalized benchmarks like “rand index” will be added for the split-merge error analysis.

## Conclusion

To obtain the 3D connection relationship from serial mitochondria and synapse segmentations, this paper proposes a fast forward 3D connection algorithm, which can be deemed as a generalization of existing Matlab’s function *bwconncomp*. The proposed method can achieve the connection performance that matches the ground truth closely. Meanwhile, it can significantly reduce the computational burden and alleviate the memory requirements compared with the function *bwconncomp*. It means that our approach can help neuroscientists to accurately and quickly obtain the meaningful statistics of mitochondria and synapses, which will greatly facilitate the data analysis of neurobiology research. To our knowledge, such method is the first work in this topic.

## Abbreviations

ATP: Adenosine triphosphate
ATUM-SEM: Automated tape-collecting ultramicrotome scanning electron microscopy
EM: Electron microscopy
FIB-SEM: Focused ion beam scanning electron microscopy
IoU: Intersection-over-Union

## Footnotes

1
http://95.163.198.142/MiRA/mitochondria31/

2
http://95.163.198.142/MiRA/synapse178/

3
https://github.com/WeifuLi/A-Fast-Forward-Connection-Algorithm
